# Serological cross-sectional studies on salmonella incidence in eight European countries: no correlation with incidence of reported cases

**DOI:** 10.1186/1471-2458-12-523

**Published:** 2012-07-16

**Authors:** Gerhard Falkenhorst, Jacob Simonsen, Tina H Ceper, Wilfrid van Pelt, Henriette de Valk, Malgorzata Sadkowska-Todys, Lavinia Zota, Markku Kuusi, Cecilia Jernberg, Maria Cristina Rota, Yvonne THP van Duynhoven, Peter FM Teunis, Karen A Krogfelt, Kåre Mølbak

**Affiliations:** 1 Division of Epidemiology, Statens Serum Institut, Copenhagen, Denmark; 2 Department of Microbiological Diagnostics, Statens Serum Institut, Copenhagen, Denmark; 3 Centre for Infectious Disease Control, National Institute for Public Health and the Environment (RIVM), Bilthoven, The Netherlands; 4 Infectious Diseases Department, Institut de Veille Sanitaire, Saint Maurice, France; 5 Department of Epidemiology, National Institute of Public Health – National Institute of Hygiene, Warsaw, Poland; 6 National Center for Surveillance and Control of Communicable Diseases, National Institute of Public Health, Bucharest, Romania; 7 National Institute for Health and Welfare, Helsinki, Finland; 8 Department of Preparedness, Swedish Institute for Communicable Disease Control (SMI), Solna, Sweden; 9 Centro Nazionale di Epidemiologia, Sorveglianza e Promozione della Salute, Istituto Superiore di Sanità, Roma, Italy; 10 Hubert Department of Global Health, Rollins School of Public Health, Emory University, Atlanta, GA, USA; 11 Department of Microbiological Surveillance and Research, Statens Serum Institut, Copenhagen, Denmark

**Keywords:** Salmonella, Europe, Epidemiology, Serology, Modelling, Surveillance, Human

## Abstract

**Background:**

Published incidence rates of human salmonella infections are mostly based on numbers of stool culture-confirmed cases reported to public health surveillance. These cases constitute only a small fraction of all cases occurring in the community. The extent of underascertainment is influenced by health care seeking behaviour and sensitivity of surveillance systems, so that reported incidence rates from different countries are not comparable. We performed serological cross-sectional studies to compare infection risks in eight European countries independent of underascertainment.

**Methods:**

A total of 6,393 sera from adults in Denmark, Finland, France, Italy, Poland, Romania, Sweden, and The Netherlands were analysed, mostly from existing serum banks collected in the years 2003 to 2008. Immunoglobulin A (IgA), IgM, and IgG against salmonella lipopolysaccharides were measured by in-house mixed ELISA. We converted antibody concentrations to estimates of infection incidence (‘sero-incidence’) using a Bayesian backcalculation model, based on previously studied antibody decay profiles in persons with culture-confirmed salmonella infections. We compared sero-incidence with incidence of cases reported through routine public health surveillance and with published incidence estimates derived from infection risks in Swedish travellers to those countries.

**Results:**

Sero-incidence of salmonella infections ranged from 56 (95% credible interval 8–151) infections per 1,000 person-years in Finland to 547 (343–813) in Poland. Depending on country, sero-incidence was approximately 100 to 2,000 times higher than incidence of culture-confirmed cases reported through routine surveillance, with a trend for an inverse correlation. Sero-incidence was significantly correlated with incidence estimated from infection risks in Swedish travellers.

**Conclusions:**

Sero-incidence estimation is a new method to estimate and compare the incidence of salmonella infections in human populations independent of surveillance artefacts. Our results confirm that comparison of reported incidence between countries can be grossly misleading, even within the European Union. Because sero-incidence includes asymptomatic infections, it is not a direct measure of burden of illness. But, pending further validation of this novel method, it may be a promising and cost-effective way to assess infection risks and to evaluate the effectiveness of salmonella control programmes across countries or over time.

## Background

Together with *Campylobacter spp.*, the non-typhoid serovars of *Salmonella enterica* subspecies *enterica* (hereafter referred to as “salmonella”) are the most commonly diagnosed bacterial cause of foodborne infections in Europe [1] and other industrialized countries, e.g. the USA [2], Canada [3], Australia [4]. Symptoms range from mild, self-limiting diarrhoea to systemic infection with fatal outcome. Acute salmonella infection may be complicated by serious sequelae, such as reactive arthritis [5]. In the USA, non-typhoid salmonella are estimated to be the leading cause of hospitalization and deaths attributable to consumption of contaminated food, causing 35% of such hospitalizations and 28% of such deaths [6].

Published data on the incidence of salmonella infections are generally based on notifications of stool culture-confirmed cases [1,2]. These cases constitute only a small fraction of all cases occurring in the community. A sequence of events must occur so that a sick person in the community gets registered as a case in a surveillance system: the person must consult a health care provider, he/she must be asked to submit a stool sample, he/she must comply with this, the stool sample must be sent to and arrive at a laboratory in satisfactory condition, it must be tested for salmonella, the test must be positive, and the positive test result must be reported. All these factors may differ considerably among countries or states, due to differences in patients’ health seeking behaviour and accessibility of health services, in clinical practices regarding stool examination, in diagnostic practices and test sensitivity in clinical laboratories. These factors are influenced by cultural, infrastructural and economic aspects and determine the degree of “underdiagnosis”. Finally, a diagnosed case will go unnoticed by the surveillance system if not reported (“underreporting”). We use the term “underascertainment” for the joint effect of underdiagnosis and underreporting. Because of the varying extent of underascertainment direct comparison of reported incidence rates from different countries or states is potentially misleading.

Little is known about the true community incidence of salmonella infections in Europe. Several studies have aimed to estimate so-called multipliers, i.e. the number of cases occurring in the community per one reported case. Community surveys of acute gastrointestinal infection (AGI) prevalence by telephone interviews were done in several countries, e.g. Norway [7], Ireland [8], Malta [9], Denmark [10], France [11], and Poland [12]. However, due to their retrospective design these studies mostly lack aetiological diagnoses and are prone to recall bias. Prospective community cohort studies of AGI including microbiological diagnostics were undertaken in England in 1993–1996 [13], in the Netherlands in 1998–1999 [14,15], and in the United Kingdom (UK) in 2008–2009 [16]. The estimated community incidence of salmonella-associated AGI was similar in the Netherlands and England (3.3 and 2.2 per 1,000 person-years, respectively) in the 1990s, but the degree of underascertainment was markedly higher in the Netherlands than in England (multipliers of 14.3 and 3.2, respectively). The recent UK study revealed a lower population incidence (0.6 per 1,000 person-years), but the higher multiplier of 4.7 indicates increasing underascertainment compared to the situation in England in 1993–1996. Due to their very high cost and demanding logistics such cohort studies cannot be easily replicated.

In the USA, community incidence of salmonella-associated AGI was estimated by combining surveillance data with information on health seeking behaviour and diagnostic practices from laboratory and population surveys in FoodNet areas [17], yielding an incidence estimate of 5.2 per 1,000 (multiplier ~39) for the period 1996–1999 [18]. For the period 2000–2008, the incidence estimate was 3.4 per 1,000 (90% credible interval [CI] 2.2-5.6) and the multiplier 29 (90% CI 18–48) [6]. Similar such “multiplier studies” in Canada and Australia showed similar results. For Canada, the estimated incidence was 2.5-6.9 per 1,000 (multiplier 13–37) in 2000–2001 [3]. For Australia, it was 2.6 (95% CI 1.5-6.2) per 1,000 (multiplier 7 [95% CI 4–16]) in 2005 [4]. However, as discussed in [4], such estimates are mostly based on extrapolations from limited data and/or expert assumptions about the various steps leading to underascertainment, resulting in large uncertainties of the estimates.

As a basis for decision making in public health and for the assessment of the health and economic burden of salmonellosis, more reliable estimates of the true community incidence of salmonella infections (and other foodborne pathogens) are highly desirable. We therefore strived to develop an alternative method of estimating the community incidence of salmonella infections, which should be affordable and independent of ascertainment artefacts, expert opinion and accuracy of interviewees’ recall. To that end, we estimated the community incidence of salmonella infections from measurements of salmonella-specific antibodies in cross-sectional sero-surveys of the general population.

We present here the results of a pilot study in eight European Union member states. We compare our so-called ”sero-incidence” estimates [19] with the incidence of salmonella cases reported through the countries’ respective surveillance systems and with published incidence estimates derived from infection risk in returning Swedish travellers [20], representing an alternative surveillance approach insensitive to differences in case ascertainment among countries.

## Methods

### Study population

Existing serum banks were identified in Denmark, Finland, France, Italy, Poland, Sweden, and the Netherlands. At least 500 serum samples were selected from these serum banks with criteria: adults (target group 18–60 years of age), sampling dates ideally covering ≥12 consecutive months, geographically representative of the sampled population. Sera had been collected over a period of 5 years (January 2003 to January 2008) with the exception of the Finnish sera, which were drawn September 2000 through March 2001. A total of 6,393 serum samples were included in the study (Table 1).

**Table 1:** Serum collections tested for antibodies against salmonella

**Country**	**Period of serum collection**	**Number of sera**	**Female-to-male ratio**	**Age [mean (range)]**
Finland	Sept. 2000 - March 2001	500	1.1	44 (30–59)
Sweden ^a^	May 2007 - Jan. 2008	525	1.7	51 (18–76)
Denmark	June 2006 - July 2007	1780	1.2	49 (18–71)
The Netherlands	Jan. 2006 - June 2007	1053	1.6	39 (18–60)
Italy	Jan. 2003 - April 2004	516	1.0	34 (18–60)
Romania	Sept. 2007	509	1.0	38 (18–60)
France	May 2003 - April 2004	1010	1.0	38 (18–60)
Poland	2004 ^b^	500	1.6	37 (18–60)

^a^ In Sweden sera from older people were included in order to achieve a sufficient sample size.

^b^ Sera from Poland were randomly chosen from a collection of sera from 2004 that had no exact sampling dates recorded.

Serum samples from Finland, Sweden, and the Netherlands were subsamples of serum banks that had been collected from the resident national population for other studies, by using probability sampling schemes to achieve best possible representativeness [21,22]. The French serum samples were from persons attending routine free health checks proposed to all adults in the general social insurance scheme, which covers >80% of the population [23]. The Danish serum samples had been collected from the resident population in parts of the capital city Copenhagen and its sub-urban and rural surroundings. In Poland and Italy, serum banks of residual sera from persons consulting the health services for a variety of reasons were used. Indications for blood draw for the Polish sera were diagnostic screening before surgical procedures (~75% of sera), health checks required for employment (~15%), miscellaneous (~10%) [24]. Italian sera originated from a previous study, where patients with acute infections or immunosuppression were excluded as per study protocol [25].

In Romania no suitable serum bank was found. Therefore serum samples were prospectively collected in one district from each of the country’s eight provinces from people attending the district medical services for reasons unrelated to AGI during September 2007.

### Antibody measurement

Serum samples were analysed for antibodies against salmonella lipopolysaccharide (LPS) antigens with an in-house ELISA using commercially available LPS antigens (SIGMA, Copenhagen) of the two most common human serovars in Europe, namely *S.* Enteritidis (O-antigens 1,9,12) and *S.* Typhimurium (O-antigens 4,5,12) as capture antigen in the solid phase. Initial attempts to develop serovar-specific ELISAs showed that there was extensive cross-reactivity between the two serovars. Therefore we developed a mixed ELISA with a 1:1 mixture of both antigens [26]. The mixed ELISA was validated by testing 964 serum samples from patients with stool culture-confirmed infections with *S.* Enteritidis or *S.* Typhimurium, and 300 healthy blood donors as reference group.

In each serum sample, immunoglobulin A (IgA), IgG and IgM was measured separately, as described in [26]. Ig concentrations were expressed in arbitrary units of optical density (OD). All serum samples were analyzed at the Department of Microbiological Surveillance and Research at Statens Serum Institut, Copenhagen, to exclude inter-laboratory variability.

### Surveillance data and data from other studies

Numbers and incidence rates of reported culture-confirmed cases of salmonella, corresponding to the period of serum collection, were directly extracted from national surveillance databases by the authors. Because of the short period of serum collection in Romania, we calculated an annualized incidence rate from case reports in the two preceding months in order to compensate for seasonal incidence fluctuations. In France and the Netherlands, there is no mandatory notification of salmonella infections. Reported incidence rates were adjusted for estimated population coverage of the respective salmonella sentinel surveillance systems of 50% (France) and 64% (the Netherlands).

We also compared our sero-incidence estimates with estimates of salmonella infection incidences from a study of infection risks in Swedish citizens returning from travel to the respective country [20].

### Statistical analysis

For each country, we calculated median OD values by Ig isotype and estimated the incidence of salmonella infections based on the serological results. This was done with a Bayesian backcalculation model, which we have described in detail previously [19]. In brief, the model is based on the kinetics of IgG, IgM, and IgA observed during a 18-month follow-up study with repeated bleeding of 302 adult Danish patients with stool culture-confirmed salmonella infections. Ig concentration is modelled as a function of time since infection, taking into account observed inter-individual variations of the antibody response.

In a “reverse” application, the model generates a probability distribution of the likely time-since-last-infection for a given set of IgA, IgG and IgM values measured in any single serum sample. The individual estimates of time-since-infection for each serum sample in the cross-sectional surveys were converted to an estimate of the annual infection incidence (“sero-incidence”) in the sampled population; point estimates and 95% credible intervals are reported. The sample size of ≥500 sera from each country resulted from simulation runs of the backcalculation model, which showed that precision of the estimates decreased rapidly with smaller sample sizes.

By dividing the sero-incidence estimates by the incidence of reported cases, we calculated multipliers, which indicate how many infections have likely occurred in the population per case reported through routine public health surveillance.

Spearman rank test was used to examine the correlation between sero-incidence and reported incidence, as well as between sero-incidence and incidence estimates derived from infection rates in Swedish travellers. P-values <0.05 were considered statistically significant.

### Ethical considerations

The existing serum banks in Denmark, Finland, France, Poland, Sweden, and The Netherlands had been established for research purposes with corresponding ethics committee approvals. The sera from Italy and Romania were left-over sera from blood samples taken for diagnostic purposes. Patients had consented to the use for research purposes; a formal ethics committee approval was deemed unnecessary by the responsible public health institutes. All serum samples were anonymised.

## Results

### Sero-incidence estimates

**Figure 1:**
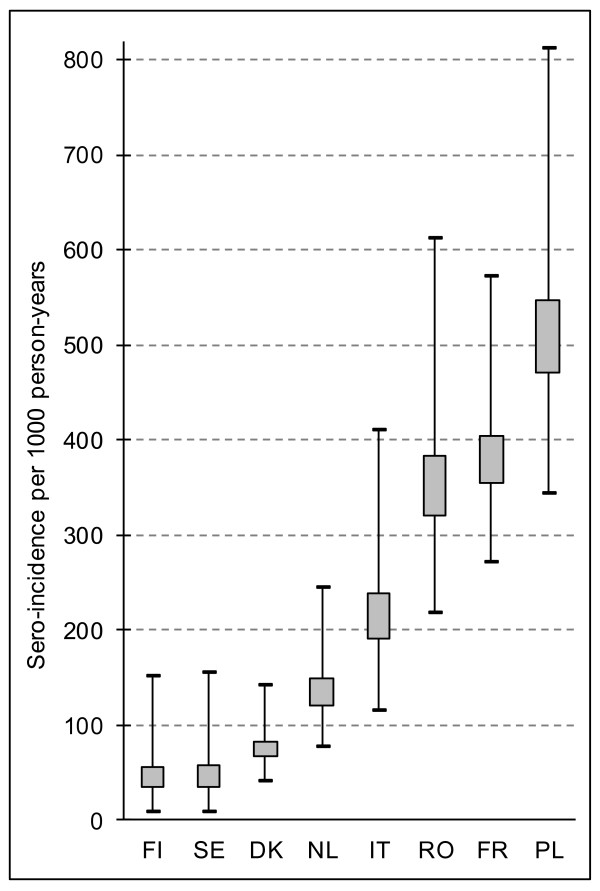
**Salmonella sero-incidence estimates in eight European countries.** Incidence of salmonella infections modeled on the basis of antibody concentrations against *Salmonella*-LPS, measured by in-house mixed ELISA in 6,393 serum samples collected between 2000 and 2008. Box: 25th and 75th percentile. Whiskers: 2.5th and 97.5th percentile. DK = Denmark, FI = Finland, FR = France, IT = Italy, NL = The Netherlands, PL = Poland, RO = Romania, SE = Sweden.

The main outcome of our study is the country-specific sero-incidence estimates (Table 2, Figure 1). Finland and Sweden had the lowest sero-incidences of 56 (95% CI 8–151) and 58 (95% CI 8–155) infections per 1,000 person-years, respectively. This is equivalent to one infection per person approximately every 17 years, whereas the highest sero-incidence of 547 (95% CI 343–813) infections per 1,000 person-years in Poland corresponds to approximately one infection per person every second year. The relative order of countries by median Ig concentration (regardless of Ig isotype) tended to be the same as for sero-incidence. In countries with low sero-incidence and low Ig values (Finland, Sweden, Denmark), ODs for IgG were lower than ODs for IgM, whereas in countries with higher sero-incidences (The Netherlands, Italy, Romania, France, Poland), ODs for IgG were higher than ODs for IgM.

**Table 2:** Salmonella sero-incidence, serum immunoglobulin concentration, incidence of reported cases, and population incidence estimate derived from infection risk in Swedish travellers

**Country**	**Sero-incidence**^**a**^**(95 % CI)**	**IgG**^**b**^	**IgM**^**b**^	**IgA**^**b**^	**Reported cases**^**a**^	**Multiplier**	**Incidence estimated from risk in Swedish travellers**^**a**^	**Multiplier**
	(A)				(B)	(A/B)	(C)	(A/C)
Finland	56 (8–151)	0.05	0.09	0.03	0.55	102	0.08	140^c^
Sweden	58 (8–155)	0.06	0.10	0.02	0.43	134	n/a	n/a
Denmark	84 (41–141)	0.07	0.10	0.04	0.29	289	0.81	104
The Netherlands	149 (78–245)	0.17	0.11	0.04	0.14	1064	0.98	152
Italy	239 (115–411)	0.24	0.14	0.06	0.12	1992	2.71	88
Romania	385 (217–613)	0.32	0.19	0.07	0.04	9625	14.57	26
France	404 (272–573)	0.25	0.21	0.07	0.20	2010	1.78	227
Poland	547 (343–813)	0.36	0.20	0.09	0.42	1302	16.26	33

^a^ per 1000 person-years.

^b^ median serum immunoglobulin concentration in arbitrary units of optical density.

^c^ adjusted for proportion of domestically acquired infections (~20%).

CI = credible interval.

n/a = not applicable.

### Comparison with other incidence data

**Figure 2:**
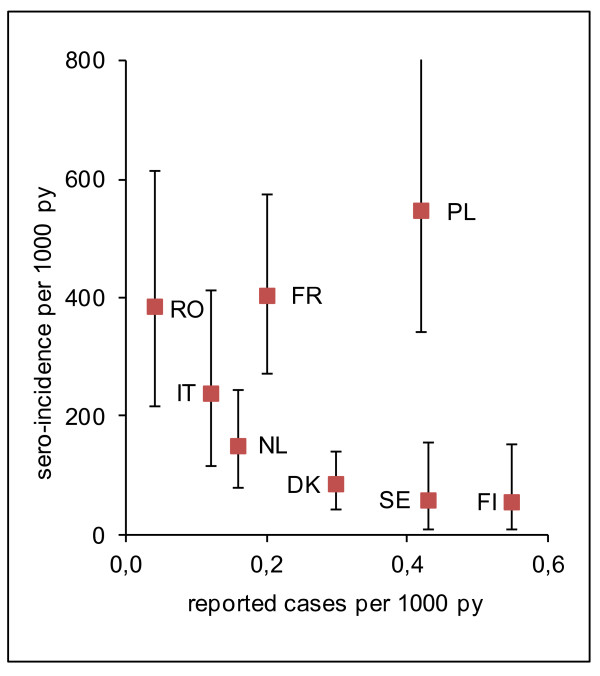
**Salmonella sero-incidence and incidence of reported cases.** Spearman’s rho = −0.5, p = 0.2. Vertical bars: 95% credible intervals py = person-years. DK = Denmark, FI = Finland, FR = France, IT = Italy, NL = The Netherlands, PL = Poland, RO = Romania, SE = Sweden.

Sero-incidence and incidence of reported cases showed a trend towards an inverse correlation (Spearman’s rho = −0.5, p = 0.2; Figure 2). Among the three countries with the highest incidences of reported cases were those with the lowest (Finland, Sweden) as well as with the highest (Poland) sero-incidence. The multipliers between sero-incidence and incidence of reported cases ranged from 102 for Finland to 9,625 for Romania. Considering Romania an outlier due to its exceptionally low reported incidence, the multipliers varied by a factor of ~20 (range 102–2,010) and tended to increase with increasing sero-incidence.

**Figure 3:**
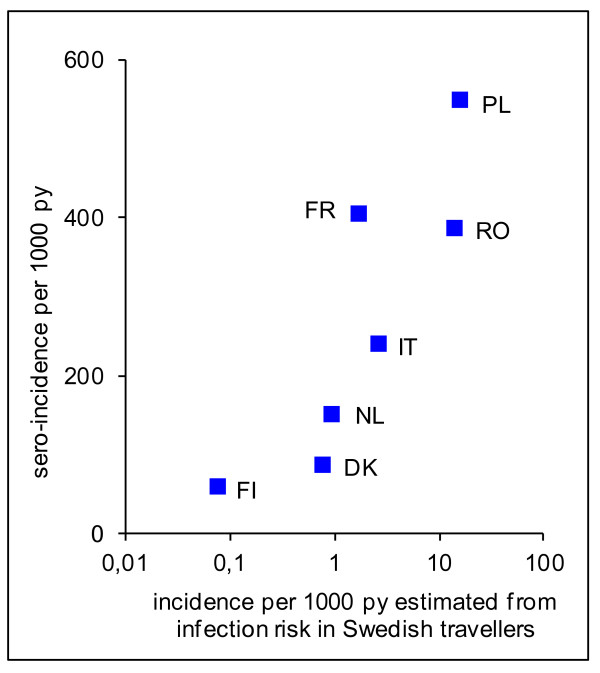
**Salmonella sero-incidence and population incidence estimates derived from infection risks in Swedish travellers.** Spearman’s rho = 0.9, p = 0.007 py = person-years. DK = Denmark, FI = Finland, FR = France, IT = Italy, NL = The Netherlands, PL = Poland, RO = Romania. Population incidence estimates derived from infection risks in Swedish travellers as reported in [20].

Incidence estimates derived from infection risks in Swedish travellers returning from the respective countries showed a statistically significant positive correlation with sero-incidence (Spearman’s rho = 0.9, p = 0.007). (Table 2, Figure 3). For Finland, the crude ratio of sero-incidence and estimate from Swedish traveller infection risk was 700. However, only ~20% of reported salmonella infections in Finland are domestically acquired [27]. Because only the domestic infection risk determines the incidence in Swedish travellers to Finland, an adjusted multiplier of 140 (20% of 700) was included in Table 2. With that adjustment, sero-incidences exceeded the incidences estimated from infection risk in Swedish travellers to the respective countries by a factor of 26 to 227.

## Discussion

We estimated the incidence of human salmonella infections in eight European Union member states, using a novel method based on cross-sectional sero-surveys and a Bayesian backcalculation model [19]. These so-called sero-incidences differed widely among participating countries from 56 to 547 infections per 1,000 person-years. Sero-incidence estimates exceeded incidences of culture-confirmed cases reported through routine surveillance by a factor of ~100 to ~2,000, depending on country.

Sero-incidence was not correlated with incidence of reported cases. If anything, there was a trend towards an inverse correlation, albeit not statistically significant. Interestingly, the lowest sero-incidences were found in Finland and Sweden, which both report higher incidences of culture-confirmed cases through their regular surveillance systems than the other six countries. These findings are compatible with the active salmonella control programmes in both countries [28,29], resulting in low infection rates in humans (as supported by serological results) and high proportion of case ascertainment. Nevertheless, sero-incidence was still ~100-130-fold higher than reported incidence.

### Limitations

The mixed ELISA used to measure serum antibody concentrations is based on LPS antigens from *S.* Enteritidis (serogroup D1) and *S.* Typhimurium (serogroup B). These two serovars comprised 75-90% of reported salmonella cases in the European Union in 2004–2007 [30-32]. It is reasonable to assume that serum from people infected with other salmonella serovars with shared LPS antigens (i.e. belonging to serogroups B or D) will be reactive in our ELISA, but we have no information about possible cross-reactivity with antibodies to salmonella from other serogroups. When calculating the multiplier between sero-incidence and incidence of reported cases, we used the number of all reported cases, irrespective of serovar. Therefore the multipliers may be underestimations.

The serum samples were from adults only, whereas the incidence of reported cases included all age groups. Because reported incidence is generally higher in young children, this was another factor contributing to underestimation of the multipliers.

The source of serum samples differed among countries. In Finland, Sweden, and the Netherlands, the sampling frame was the entire national resident population. In Denmark it was the resident population in one region, whereas in France, Poland, Romania, and Italy serum samples had been collected from persons consulting the health services for health checks or for illnesses unrelated to acute gastroenteritis. These four countries also have the highest sero-incidence estimates. It can be speculated that the estimates may be biased, because people who are willing to use and have good access to formal health services were more likely to be sampled. However, this would only bias the sero-incidence estimates upwards if the risk of salmonella infection (diagnosed or not) in this group was higher than in the general population. For people attending for health checks this seems unlikely, unless persons with undeclared AGI or immunocompromising conditions were included in the study in relevant numbers. The validity of the high sero-incidence estimates is supported by the fact that also infection risk in Swedish travellers is highest in the same four countries. Future studies should investigate what kind of sera could replace truly population-representative serum collections, which are non-existant or not accessible in many countries.

The larger sample size in some countries, including older people in Denmark, allowed us to analyse the effect of age, gender, and sampling month on the sero-incidence estimates in a multivariate regression model. None of these three factors significantly influenced sero-incidence estimates [33]. Therefore, we did not exclude sera from older people or from countries with serum collection during less than a whole year.

Finally, the backcalculation model is based on data of antibody decay over time that was observed in salmonellosis patients in Denmark, a country with a relatively low incidence of salmonella infections. It is difficult to predict how this may have affected the sero-incidence estimates for high incidence countries. If the antibody response is stronger with frequent infections, our model would overestimate infection incidence. However, if frequent infections induce a weaker immune response, especially lower IgM production, our model would underestimate infection incidence.

### Comparison with other data

It was reassuring that, despite these limitations, the sero-incidence estimates were correlated with the incidence estimates derived from infection risk in returning Swedish travellers. It should be noted that the infection risk in Swedish travellers reflects only the risk of domestic salmonella transmission in the visited country, whereas sero-incidence includes both domestic and imported infections. This is particularly relevant in countries with low incidence of domestic infections and thus a relatively large proportion of imported infections.

How do our findings compare with data from other studies attempting to estimate the population incidence of salmonella-associated AGI? Based on data from the Dutch prospective SENSOR community cohort study [14], and adjusted for the trend in reported cases, Kemmeren et al. [34] calculated a population incidence of 2.2 per 1,000 persons in the Netherlands in 2004. In contrast, our sero-incidence estimate for the Netherlands is 149 salmonella infections per 1,000 person-years, about 70 times higher.

This discrepancy can at least partially be explained by the fact that the SENSOR study counted episodes of clinical gastroenteritis whereas sero-incidence includes all infections inducing a sero-response, including those with mild or possibly no symptoms (“subclinical infections”). Therefore the multiplier between reported incidence and sero-incidence is a compound indicator of the underascertainment of symptomatic cases through surveillance and the ratio of illness episodes to subclinical infections (“disease-to-infection ratio”) in the population. We cannot directly estimate this ratio because we do not have information on disease history from the serum donors in our study. It is likely that the proportion of subclinical infections increases with increasing sero-incidence, reflecting partial immunity in the population when contact with salmonella is frequent. In addition, countries with high salmonella incidence may also have less rigorous case ascertainment. A combination of both effects likely explains our observation that the multiplier between sero-incidence and reported incidence increased with increasing sero-incidence (Table 2).

To our knowledge salmonella-specific multiplier studies, as performed in the USA, Canada and Australia [3,4,6,18], have not been done in Europe. The mentioned studies yielded estimates of the population incidence of salmonella-associated AGI in the range of approximately 2 to 6 per 1,000 person-years. The ratios of the upper and lower limits of the 95% credible intervals, if reported, were approximately 2.5 to 4. This degree of uncertainty is very similar to the uncertainty of our sero-incidence estimates, with the exception of Finland and Sweden, where the sero-incidence was so low that the sample size of only 500 sera resulted in very large credible intervals.

### Perspectives

To further validate our method and inform interpretation of the sero-incidence estimates, several additional studies should be done. It should be determined what proportion of all salmonella infections is sub-clinical, how that depends on the overall infection incidence, and how antibody response after subclinical infection differs from antibody response after illness. Longitudinal serological follow-up of salmonellosis patients should be repeated in a high incidence country to investigate how the antibody response is affected by frequent exposure to salmonella. Antibody decay should also be studied in children, because their immune response likely differs from that in adults. This would be a prerequisite for sero-incidence estimation from cross-sectional sero-surveys in children. Sera from patients infected with other salmonella serovars should be tested with our ELISA to check if they cross-react.

To facilitate replication of sero-incidence studies in other settings, it should be studied if truly population-representative serum collections can be substituted by more readily accessible serum samples, for instance from blood donors, orthopaedic patients, or from screening programmes in pregnancy. Even though such sera may not be representative for the entire population, they would be suitable for comparing incidences of salmonella infections among countries, because a possible bias should be similar in all countries.

## Conclusions

Sero-incidence estimation is a promising new method to estimate and compare the incidence of salmonella infections in human populations independent of the extent of underascertainment of cases through routine public health surveillance. Sero-incidence is not a direct measure of burden of illness, but it allows comparison of infection risks among countries - information that is valuable to assess for example the public health impact of different food safety policies. Sero-incidence can also be useful to monitor time trends of salmonella incidence and evaluate the effect of control interventions, independent of modifications of surveillance practice over time. The method can potentially be applied to other common infections, e.g. campylobacteriosis. While reported incidences can serve to monitor trend over time *within* a country, our results confirm that comparison of reported incidences *among* countries, even within the European Union, can be grossly misleading.

## Competing interests

The authors declare that they have no competing interests.

## Authors’ contributions

GF coordinated implementation of the study, contributed to data collection, analysis and interpretation, and wrote the manuscript. JS and PFMT performed the statistical analyses. THC and KAK performed the serologic tests and provided sera from Denmark. WvP, HdV, MST, LZ, MK, CJ, MCR, YTHPvD provided sera and surveillance data from their respective countries. In addition, all authors were involved in the study design, data interpretation and reviewing several versions of the manuscript. All authors had full access to all study data. GF and JS had final responsibility for the decision to submit for publication. All authors read and approved the final manuscript.

## Pre-publication history

The pre-publication history for this paper can be accessed here:

http://www.biomedcentral.com/1471-2458/12/523/prepub
